# Supplementary material for: Preparation of Gold Nanoparticles via Anodic Stripping of Copper Underpotential Deposition in Bulk Gold Electrodeposition for High-Performance Electrochemical Sensing of Bisphenol A
Source: Molecules. 2023 Dec 11;28(24):8036. doi: 10.3390/molecules28248036 (PMC10745752; doi:10.3390/molecules28248036)
Supplement: Supplementary file 1 [file molecules-28-08036-s001.zip › molecules-2689429-supplementary.pdf]

Supporting Information For

Preparation of Au nanoparticles *via* anodic stripping of underpotential deposition-Cu in bulk electrodeposition-Au for high-performance electrochemical sensing of bisphenol A

Zhao Huang [1], Zihan Chen [1], Dexuan Yan [1], Shuo Jiang [1], Libo Nie [1], Xinman Tu [2], Xueen Jia [1,3], Thomas Wågberg [3] and Long Chao [1,*]

[1] Hunan Key Laboratory of Biomedical Nanomaterials and Devices, College of Life Sciences and Chemistry, Hunan University of Technology, Zhuzhou 412007, China
[2] Key Laboratory of Jiangxi Province for Persistent Pollutants Control and Resources Recycle, Nanchang Hangkong University, Nanchang 330063, China
[3] Department of Physics, Umeå University, SE-901 87, Umeå, Sweden
*Correspondence: E-mail: chaolong4617@163.com

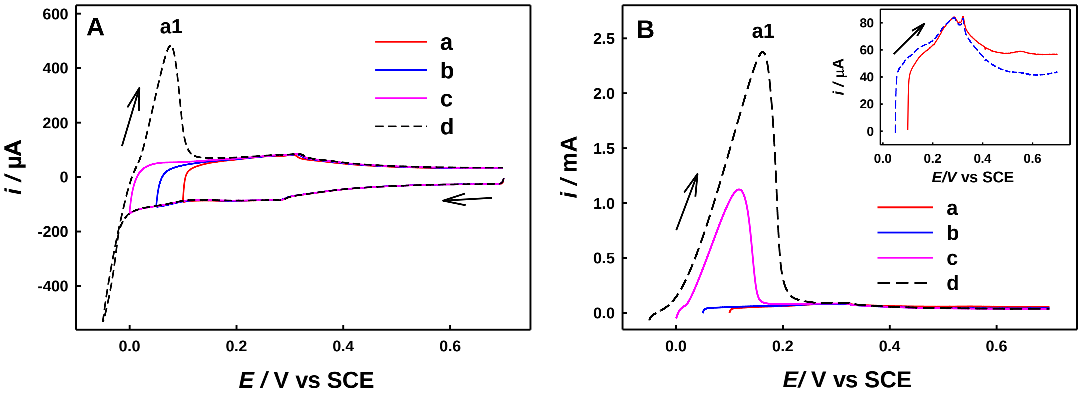

Figure S1. (A) CV curves with different negative potential limits (a: 0.1 V, b: 0.05 V, c: 0 V and d: –0.05 V) and (B) LSASV curves on Au electrode after 20 s preconcentration at 0.1 V (a), 0.05 V (b), 0 V (c) and –0.05 V(d). Inset: magnification of LSASV curves a and b for the graph B. Solution: 10 mM $CuSO_4$ + 0.2 M aqueous $HClO_4$. Scan rate: 50 mV s^{-1}. The arrow indicates the direction of the scanning here.

The CV experiments show that the oxidation peak of Cu(0) to Cu^{2+} (P_{a1}) appears for a negative potential limit at –0.05 V (d). However, at negative potential limit of 0.1 V (a), 0.05 V (b) and 0 V (c), the oxidation peak of Cu(0) to Cu^{2+} (P_{a1}) disappears. The LSASV curves after preconcentration for 30 s show that peaks P_{a1} appear when the preconcentration potential is at –0.05 V (d) and 0 V (c), but peak P_{a1} disappear after preconcentration at 0.05 V (b) and 0.1 V (a). The small oxidation peaks indicate the anodic stripping of UPD-Cu in the inset. The oxidation peak of Cu(0) to Cu^{2+} (P_{a1}) disappears by CV but appears by LSASV at negative potential limit of 0 V here, which could be due to a short time for BD of Cu by CV. In general, both CV and LSASV experiments indicate that here only UPD of Cu but no BD of Cu occur at 0.05 V and 0.1 V. In oder to completely avoid the simultaneous BD of Cu on Au, a deposition potential of 0.1 V was selected here for preparation of $Au_{Cu\text{-}UPD}$/MWCNTs/GCE. In addition, the anodic stripping of UPD-Cu can completely finished at 0.6 V as shown in the inset of graph B.

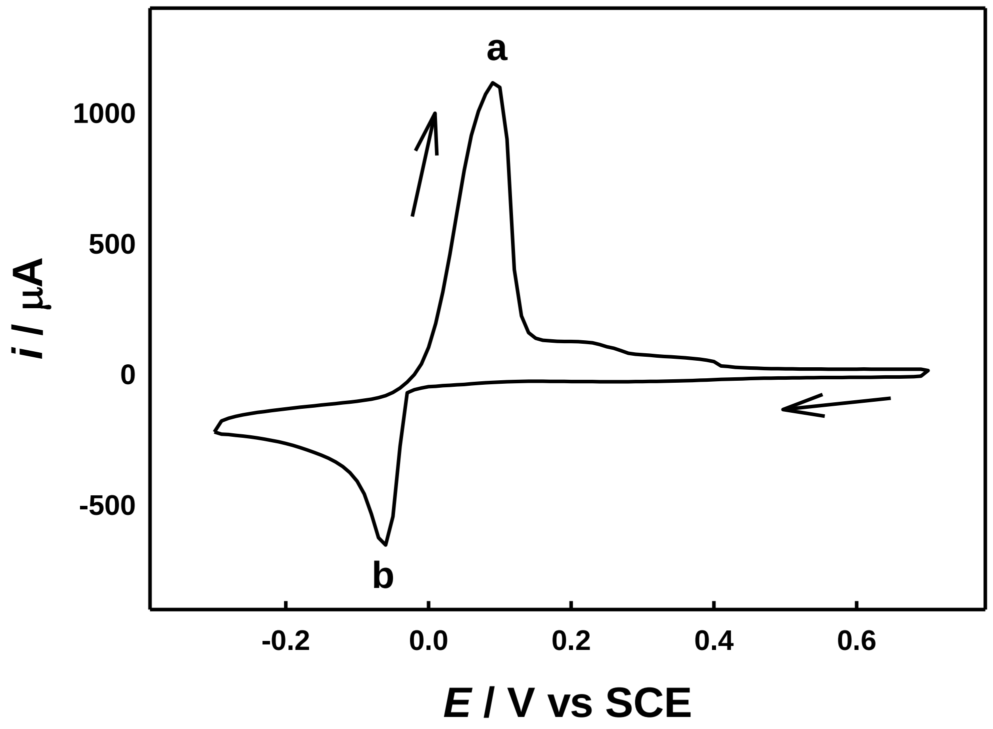

Figure S2. CV curves on the Au_{Con}/MWCNTs/GCE in 0.2 M aqueous $HClO_4$ containing 10 mM $CuSO_4$. Scan rate: 50 mV s^{-1}

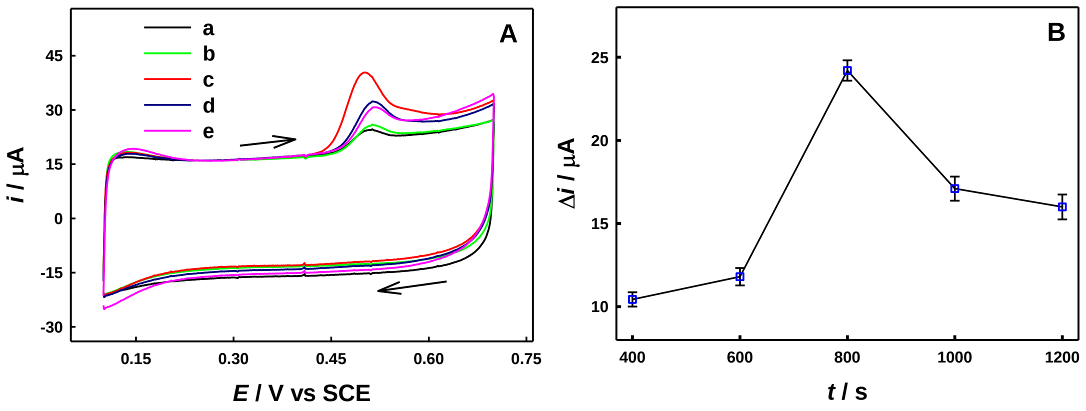

Figure S3. CV curves of the $Au_{Cu\text{-}UPD}$/MWCNTs/GCEs prepared by varying Cu-underpotential/Au-bulk coelectrodeposition time in 0.1 M PBS (pH 7.5) containing 10 μM BPA (A), and relationship between the oxidation peak current of BPA and coelectrodeposition time. Scan rate: 50 mV s^{-1}.

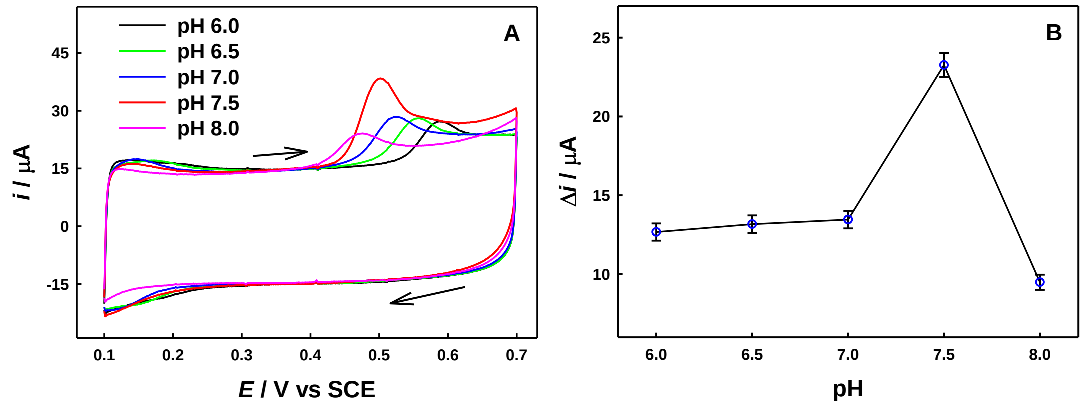

Figure S4. CV curves of the $Au_{Cu\text{-}UPD}$/MWCNTs/GCEs in 0.1 M PBS with different pH values (6.0, 6.5, 7.0, 7.5 and 8.0) containing 10 μM BPA (A), and a plots of peak current against pH (B). Scan rate: 50 mV s^{-1}.

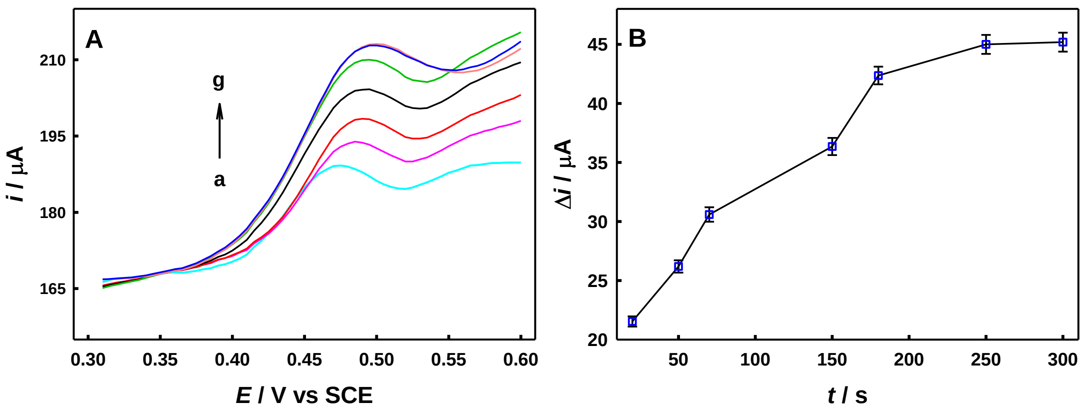

Figure S5. DPV curves of the $Au_{Cu\text{-}UPD}$/MWCNTs/GCE in 0.1 M PBS (pH 7.5) containing 10 μM BPA at different preconcentration time (A) and a plots of peak current of the corresponding DPV curves against different preconcentration times (B). Preconcentration potential: 0.3 V.

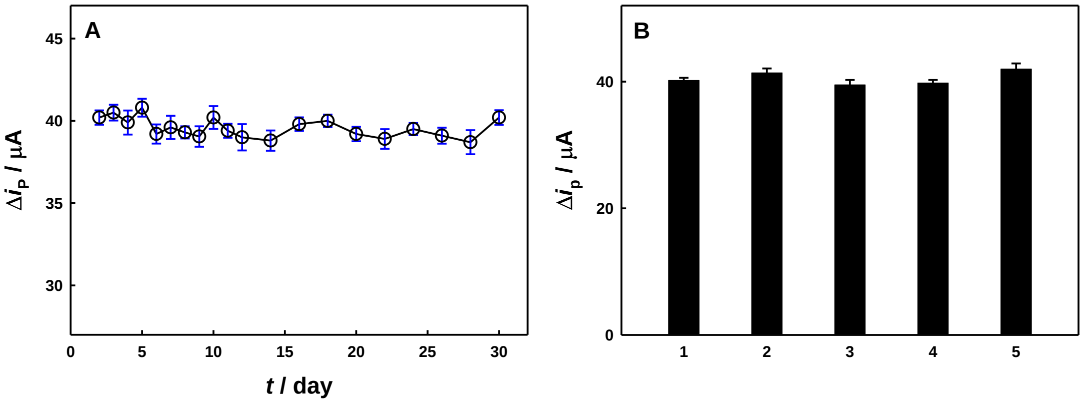

Figure S6. Stability of $Au_{Cu\text{-}UPD}$/MWCNTs/GCE with repeated detection of BPA for 30 days (A). Reproducibility on five $Au_{Cu\text{-}UPD}$/MWCNTs/GCE (No.1-5) with detection of BPA (B). All date were obtained from every DPV response in 0.1 M PBS (pH 7.5) containing 5 μM BPA and average values calculated from three determinations. 300 s preconcentration and at preconcentration potential of 0.3 V.
